# Supplementary material for: A Novel Class of Human ADAM8 Inhibitory Antibodies for Treatment of Triple-Negative Breast Cancer
Source: Pharmaceutics. 2024 Apr 13;16(4):536. doi: 10.3390/pharmaceutics16040536 (PMC11054802; doi:10.3390/pharmaceutics16040536)
Supplement: Supplementary file 1 [file pharmaceutics-16-00536-s001.zip › pharmaceutics-2917108-supplementary.pdf]

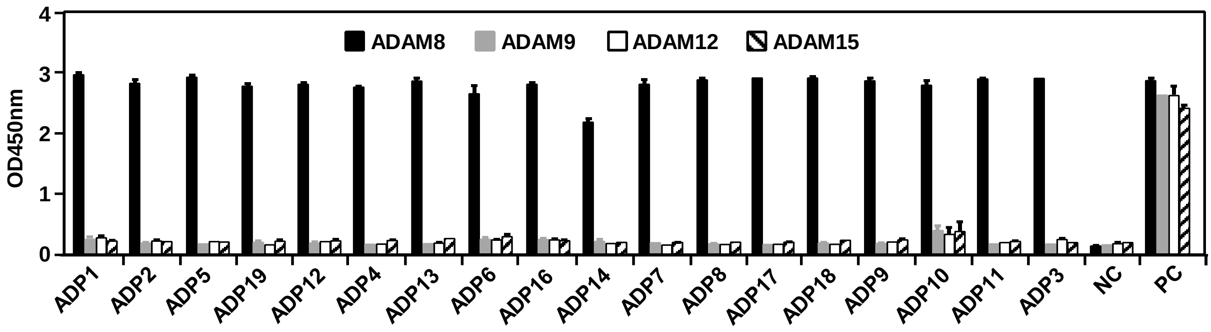

Supplementary Figure S1. ADPs selectively bind ADAM8. ELISAs were performed using recombinant human proteins of ADAM8 closely related ADAM9, ADAM12 or ADAM15 proteins to assess cross-reactivity. Normal mouse IgG was used as negative control (NC); a test bleed sample from a recombinant human ADAM8 (rHuADAM8) injected mouse was used as a positive control (PC).

Epitope	Ab	ADP10	ADP17	ADP18	ADP19	ADP11	ADP8	ADP5	ADP13	ADP2	ADP1	ADP12	ADP7	ADP9	ADP6	ADP4	ADP3	ADP16	ADP14
	ADP10	91%	90%	88%	82%	91%	90%	59%	44%	24%	26%	16%	37%	47%	68%	49%	14%	45%	-13%
	ADP17	90%	93%	88%	90%	94%	92%	45%	65%	36%	52%	42%	47%	57%	63%	53%	32%	35%	-26%
	ADP18	91%	94%	90%	89%	94%	91%	32%	49%	30%	21%	13%	34%	50%	60%	46%	7%	45%	-29%
1	ADP19	90%	93%	91%	88%	92%	92%	79%	37%	-4%	16%	3%	26%	31%	34%	33%	0%	25%	-24%
	ADP11	91%	93%	90%	88%	94%	92%	82%	47%	10%	-3%	9%	48%	50%	46%	24%	3%	37%	5%
	ADP8	92%	92%	88%	84%	93%	91%	89%	44%	2%	8%	4%	17%	37%	42%	20%	6%	25%	-27%
	ADP5	91%	93%	92%	86%	94%	94%	81%	54%	14%	26%	5%	42%	22%	37%	33%	4%	54%	-30%
2	ADP13	52%	56%	23%	66%	27%	46%	27%	92%	86%	80%	89%	-9%	-3%	19%	47%	3%	12%	-18%
	ADP2	26%	26%	11%	10%	13%	11%	1%	94%	91%	83%	90%	91%	85%	93%	94%	93%	41%	-10%
	ADP1	24%	7%	12%	6%	13%	9%	3%	89%	91%	78%	90%	88%	85%	94%	95%	95%	34%	1%
	ADP12	31%	18%	7%	42%	6%	6%	-17%	94%	90%	85%	90%	83%	79%	85%	89%	80%	22%	-25%
3	ADP7	64%	61%	40%	48%	54%	48%	87%	33%	66%	77%	60%	87%	83%	91%	88%	73%	86%	-29%
	ADP9	58%	61%	34%	49%	39%	42%	68%	52%	81%	83%	79%	88%	84%	87%	89%	84%	90%	-30%
	ADP6	40%	38%	17%	57%	16%	30%	-5%	22%	76%	76%	69%	89%	84%	93%	95%	78%	87%	6%
	ADP4	35%	35%	9%	28%	11%	25%	3%	32%	61%	76%	53%	85%	82%	91%	92%	66%	84%	-25%
	ADP3	34%	16%	15%	15%	15%	-1%	6%	31%	89%	80%	89%	89%	87%	93%	92%	93%	89%	6%
4	ADP16	35%	7%	7%	26%	12%	-2%	-3%	47%	43%	56%	34%	79%	81%	89%	86%	72%	88%	-7%
5	ADP14	46%	32%	19%	39%	27%	6%	15%	40%	40%	62%	13%	60%	70%	83%	76%	57%	20%	82%

Supplementary Figure S2. ADPs have 5 epitope clusters on the ectodomain of ADAM8. Epitope binning was performed using competitive ELISA. The binding of each ADP [indicated in the "Ab" column] to rHuADAM8 was challenged with excess of a second competitor ADP (indicated in the top row of the table). Percentages indicate extent of cross-competition for ADAM8. High levels of cross-competition, defined as equal to or greater than 75% (marked by the darker green shades), were used to delineate epitope clusters on ADAM8 for ADP binding. White boxes are the values obtained for competition with self.

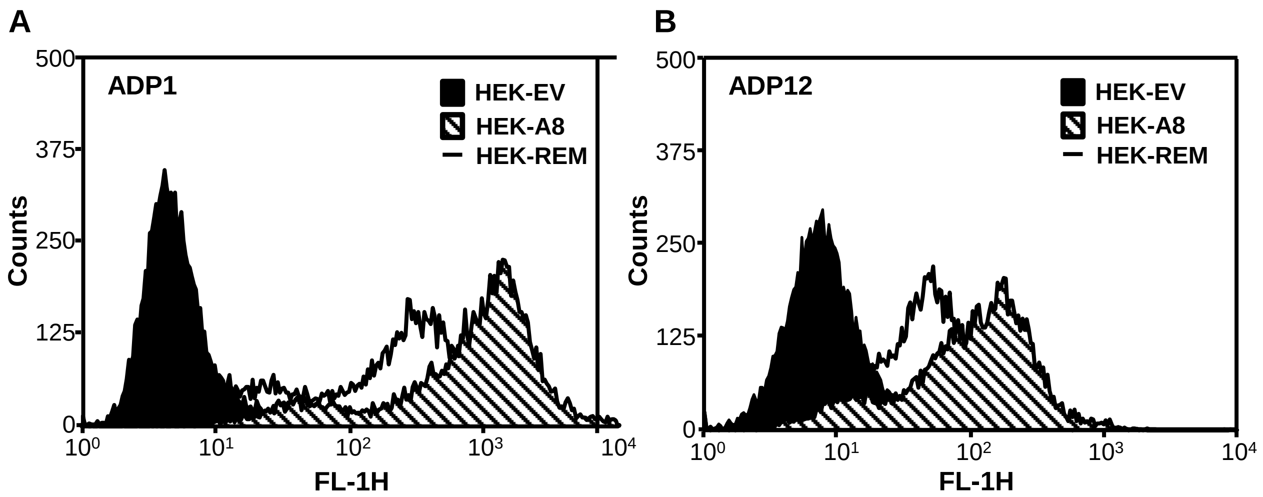

Supplementary Figure S3. Epitope 3 Abs ADP1 and ADP12 bind to both full-length and Remnant ADAM8**.** HEK293 cells stably transfected with a plasmid expressing full-length ADAM8 (HEK-A8), a deletion construct which generates the Remnant form, lacking the PRO and MP domains (HEK-REM) or empty vector control DNA (HEK-EV) were stained with ADP1 (**A**) or ADP12 (**B**), and subjected to flow cytometry. Representative histograms are shown.

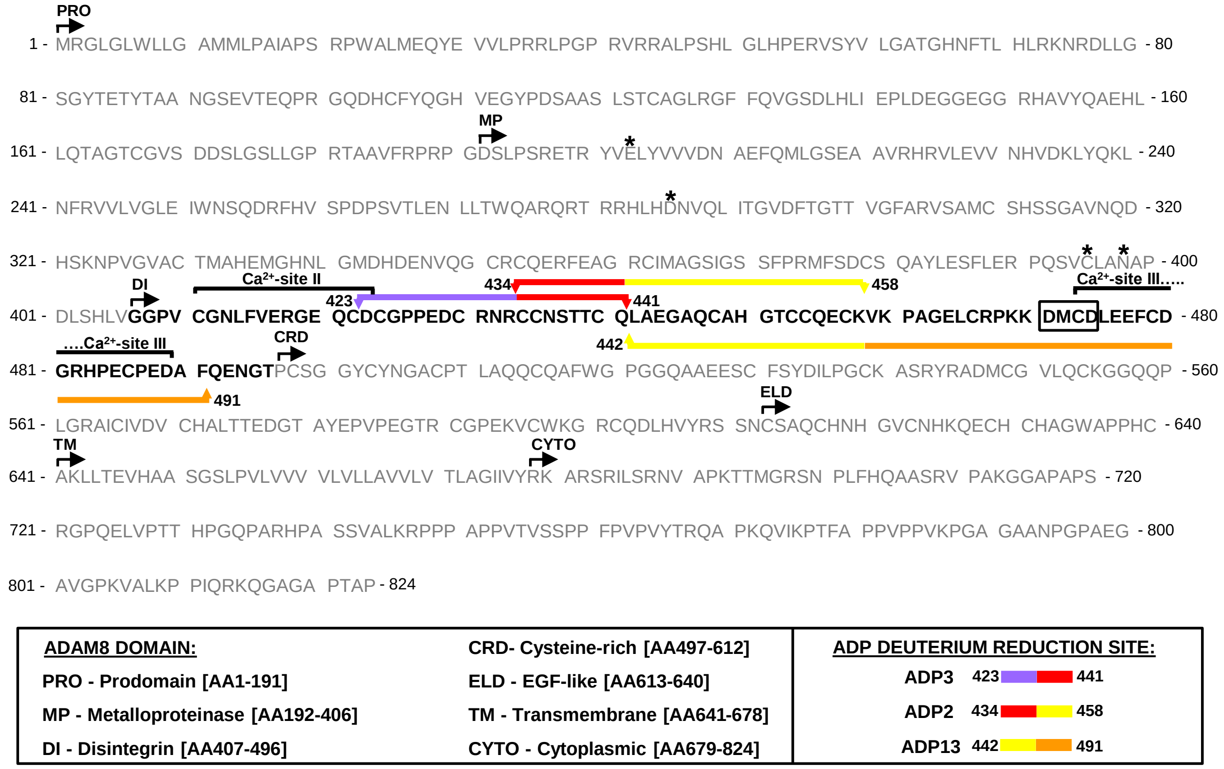

Supplementary Figure S4. Diagram of the human ADAM8 sequence with its domain regions indicating the epitopes of ADP2, ADP3 and ADP13 binding within the DI, at the peptide level as identified by HDX-MS. Calcium ion binding site I (involving 4 starred AAs in the MP), and sites II and III in the DI are as indicated. The integrin binding sequence (DMCD, AA471-474, open box), stabilized by disulfide bonds and calcium binding to adjacent site III, is shown. The human ADAM8 GenBank number is AAI15405.1.

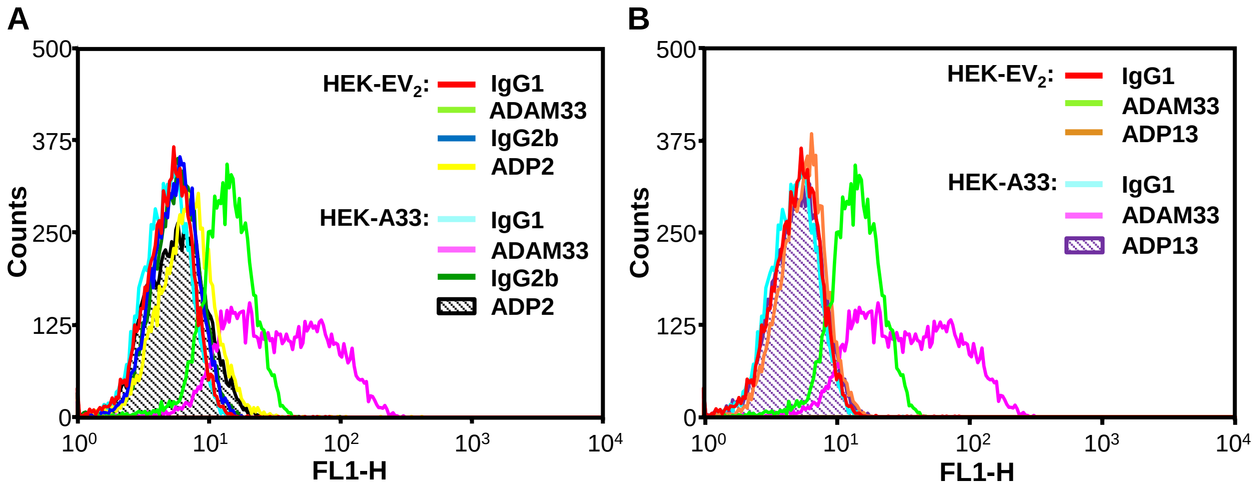

Supplementary Figure S5. ADP2 and ADP13 do not cross-react with ADAM33**.** HEK293 cells transiently transfected with a plasmid expressing ADAM8 related protein ADAM33 (HEK-A33) or empty vector control DNA (HEK-EV_2) were stained with ADP2 (**A**) or ADP13 (**B**), following permeabilization to access the cytoplasmically localized ADAM33, and subjected to flow cytometry. An ADAM33 Ab was used as a positive control and appropriate isotype-matched IgGs as negative controls. Representative histograms are shown.
